# P-TransUNet: an improved parallel network for medical image segmentation

**DOI:** 10.1186/s12859-023-05409-7

**Published:** 2023-07-18

**Authors:** Yanwen Chong, Ningdi Xie, Xin Liu, Shaoming Pan

**Affiliations:** The State Key Laboratory of Information Engineering in Surveying, Mapping, and Remote Sensing, Wuhan University, Wuhan, China

**Keywords:** Medical image segmentation, Transformer, Self-attention, Axis attention, Channel attention

## Abstract

Deep learning-based medical image segmentation has made great progress over the past decades. Scholars have proposed many novel transformer-based segmentation networks to solve the problems of building long-range dependencies and global context connections in convolutional neural networks (CNNs). However, these methods usually replace the CNN-based blocks with improved transformer-based structures, which leads to the lack of local feature extraction ability, and these structures require a huge number of data for training. Moreover, those methods did not pay attention to edge information, which is essential in medical image segmentation. To address these problems, we proposed a new network structure, called P-TransUNet. This network structure combines the designed efficient P-Transformer and the fusion module, which extract distance-related long-range dependencies and local information respectively and produce the fused features. Besides, we introduced edge loss into training to focus the attention of the network on the edge of the lesion area to improve segmentation performance. Extensive experiments across four tasks of medical image segmentation demonstrated the effectiveness of P-TransUNet, and showed that our network outperforms other state-of-the-art methods.

## Introduction

Medical imaging, such as computed tomography (CT), magnetic resonance imaging (MRI), and gastroscopy, is greatly important for clinicians to make a preliminary diagnosis of the current condition of patients [1]. However, the huge number of medical images requires several experts to process. A computer-aided diagnosis system (CADS) helps clinicians by producing the suspected lesion area or organ boundaries to make full use of medical images [2]. As a computer vision task, image segmentation can classify images at the pixel level and is promising in the field of medical imaging [3]. In application, the structure extracts semantic features of input images through an automated process and then classifies the image at the pixel level, which provides a feature-based approach for pathological studies and increases the accuracy of diagnoses in clinical practice.

With the development of deep learning, convolutional neural networks (CNNs) gradually dominate the field of image segmentation [4]. As the most classic network in medical imaging, U-Net [5] has proved its excellent performance on various types of image data. It consists of an encoder and a decoder, where the encoder extracts high-level features through convolution and down-sampling layers, and the decoder generates the result through up-sampling with skip connections, which provide details of different granularities. Benefiting from the U-shaped architecture, many novel structures have been developed and proposed in recent studies, such as U-Net++ [6], Res-UNet [7], and Dense-U-Net [8]. These networks were adjusted to generate more specific features from images based on the characteristics of the concerned areas and achieved promising success. However, CNN-based structures have their disadvantages, which hinder the development of medical image segmentation. First, because of the definition of convolution kernel, each convolution kernel can only focus on the local region of the image, which may lose global information and fail to establish the long-range relationship [9]. Second, the pooling layers may cause the network to lose critical details of the images and their inner relationship. Some studies have been conducted to collect the long-range dependency for convolutional networks, such as Atrous Spatial Pyramid Pooling (ASPP) [10] and attention [11]. Because of the unique characteristics of medical images, such limitations of the aforementioned models should be addressed to meet the requirements of medical tasks.

Transformers [12] were first proposed in natural language processing (NLP) and achieved great success in various tasks for its excellent ability to connect long-range dependency information. Furthermore, transformers were first introduced into computer vision tasks to build the network named Vision Transformer (Vit) [13], which achieves comparable performance with other convolution-based methods. Transformer models are attention-based and their key component is self-attention (SA). SA can model correlations among all input tokens with equal weights instead of focusing on the local position like CNN-based models, which makes the long-range dependencies of transformer models to get distinguishable features. However, Vit has its drawbacks: first, it requires several images for training; however, these images are limited in the field of medical imaging [14]. Second, the SA of transformer models slows down the processing speed of high-resolution images because of its quadratic computational complexity. Third, the calculation of SA reduces the attention weight of local features, which may lead to lost information on local details.

At present, many studies [9, 14–16] have combined CNN- and transformer-based models to propose novel structures. However, these studies mostly optimized the feature extraction ability in series, while ignoring the inherent influence of the structure on feature extraction. A common operation is to propose a new transformer-based structure to replace convolution layers for feature extraction or aggregation. Although such a replacement alleviates the inherent inductive biases of CNNs and enhances the ability to model global–local context information, the operation also weakens the ability to detail feature extraction because of the discard of CNNs in the encoder. In medical image segmentation, the detail texture feature plays a vital role in segmenting lesion areas [17], which can be well aggregated by convolution layers. Therefore, we proposed a new structure that integrates transformer- and CNN-based models to model detailed information and global relationships simultaneously. Studies have reported that encoders based on the two structures can extract high-level features of images simultaneously [16], and there will be some repetitive information. Thus, we adjusted the transformer structure to pay more attention to long-range dependencies and ignore local information to avoid extracting repetitive local information, which will only be extracted via the paralleled CNN model. Besides, the edge information [18] of the lesion area contains the comparison of the normal and diseased areas, which plays an important role in diagnosis. By introducing the supervision of region edges, we added edge information into the field of medical images for segmentation. The main contributions of our work are as follows:

We propose P-Transformer, an encoder structure, achieved by combining the designed transformer and convolution layers in parallel for feature extraction. The structure can integrate the advantages of both so that the network can effectively model local and global information and avoid the interference of repeated information.

To fuse the features, we propose an attention-based fusion module that integrates two types of features in the channel and spatial dimensions. Besides, we introduced edge information as supervision along the training process, which allows the network to focus on the edge details of the target area to improve performance in the medical imaging field.

We performed experiments on several medical image segmentation datasets to verify the effectiveness of our proposed network. The results showed that the proposed structure with edge loss had higher segmentation performance than previous transformer-based networks, and the visualization effect also proved the effectiveness of our method.

## Related work

In this section, we briefly summarize the current research on medical image segmentation. We first summarized the U-shaped network represented by U-Net, the most typical CNN method in medical image segmentation, and then introduced the application of visual transformers in the field of image segmentation, particularly in medical imaging tasks.

### Medical image segmentation based on CNNs

Early medical image segmentation algorithms were mainly based on edge extraction operators of contour and machine learning algorithms. Owing to the development of deep convolutional networks, U-Net [5] was developed and proposed for medical image segmentation, and demonstrated excellent segmentation performance in the medical field. Benefiting from the U-shaped structure, U-Net uses an encoder and a decoder to extract image features and introduce a skip connection to retain details. Many novel architectures based on U-Net have been proposed to improve the performance of vision tasks. U-Net++ [6] introduced multilevel dense skipping connections to further model local details to reduce high-level semantic information gaps. Res-UNet [7] introduced a residual structure and combined attention mechanism to solve the problem of topological structure and contrast in retinal vascular segmentation tasks. DoubleU-Net [19] is a combination of two U-Net architectures stacked on each other, and the first U-Net uses pretrained VGG-19 as the encoder, which can be easily transferred to another task. Besides, ASPP [10] is adopted to collect context information on images. Note that these methods are based on CNN; therefore, the convolution layer has inherent inductive bias and missing global relationship information problems. Although these problems can be improved by adding adjusted attention modules, long-distance dependence information still cannot be effectively modeled.

### Transformers in medical segmentation

Inspired by the excellent performance of transformers in NLP tasks, SA-based transformers have been introduced into computer vision and made great progress. First, Vit [13] introduced the transformer structure to replace the CNN layers in the computer vision field and achieved better performance than previous popular deep convolution networks. It cuts the image into different tokens and adds a position offset to complete the mapping from the picture to the sequence, and then extracts long-distance dependency features of the image to enhance semantic information. Swin-transformer [14] proposed a new hierarchical backbone structure that realized the linear computational complexity based on the self-attention of a sliding window and improved the segmentation performance based on reducing the calculation cost. DS-transUNet [20] proposed a new encoder–decoder-based transformer framework that combines the characteristics of Swin-transformer with multiscale visual transformers and effectively improves the standard U-shaped model structure of medical image segmentation. These structures replace the CNN structure with a transformer in encoders. Although this replacement strengthens the ability to model the long-dependency relationship of the network, it also produces a lack of detailed information and the requirements of the amount of training data. Inspired by these studies, we proposed a network that combines the structure of transformers and CNNs simultaneously, and adjusted its architecture, which not only retains the long-distance modeling ability but also reduces the calculation amount of the network to improve its trainability.

However, the aforementioned medical image segmentation models have low accuracy in medical image segmentation tasks in complex environments. The reason is that the spatial detail information of the lesion area is not fully used. Although the TransUnet model uses a transformer structure to fuse global features, it only focuses on semantic information and does not improve the acquisition process of texture features. Therefore, these models cannot fuse texture and global information simultaneously in the decoding process.

Inspired by these methods, we propose a U-shaped structure called P-TransUNet, which extracts weight in parallel by convolution and transformer, and performs feature enhancement. We believe that this parallel transformer-based structure is superior to previous serial structure models and optimizes medical image segmentation.

## Methods

**Fig. 1:**
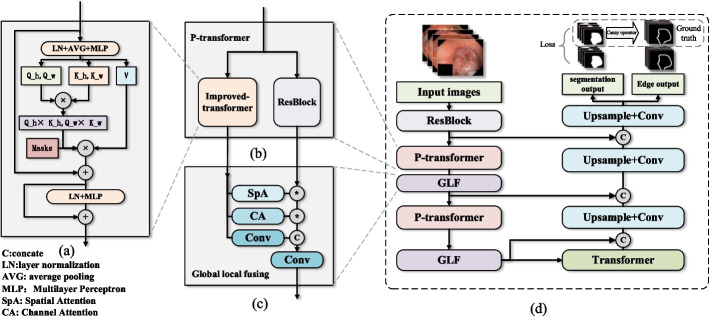
Illustration of proposed P-TransUnet and its details. **a** is the detailed diagram of improved-transformer, **b** is the detailed diagram of P-transformer, **c** is the detailed diagram of GLF modules, and **d** is the overall architecture of P-TransUnet

In this section, the overall architecture of the proposed P(parallel)-TransUNet is introduced in detail, as shown in Fig. 1. We first compared the standard transformer and the improved P-Transformer (parallel transformer) in our work, including the axial weight and weight assignment mask. Then, we introduced the global–local fusing (GLF) module for combining features produced by P-Transformer efficiently. Finally, we introduced the loss function used in the experiments, which included edge supervision to focus on salient features.

### Overview of the P-TransUNet

As shown in Fig. 1, P-TransUNet consists of an improved encoder, a CNN-based decoder, and a skip connection. When getting an image as input, the encoder first uses the former part of Resnet-50 to extract low-level features and save the outputs of each part simultaneously to prepare for skip connections. The basic unit of the encoder is P-transformer and GLF. P-transformer comprises an improved transformer and a residual network in parallel, which model the long-dependence and local information of the feature map, respectively. The outputs are fused through a GLF attention module for subsequent processing. A standard transformer is added between the encoder and the decoder to integrate features after dimension reduction. The decoder adopts a standard convolution layer and bilinear up-sampling to decode features, and reconstruct image segmentation results with the information about skip connection inputs. The detailed structures of P-transformer and GLF are introduced in the following contents.

### P-transformer

#### Standard transformer

The standard transformer [13] consists of multiple identical blocks. Each block comprises Multi-head Self-Attention (MSA) and Multi-Layer Perceptron(MLP). Furthermore, there is a Layer Norm behind each MSA and MLP with a residual connection. The output $z_{l}$ of the l-layer can be expressed as follows:

1 $$\begin{aligned} \hat{z}_{l} &= MSA\left( {LN\left( {z_{l - 1} } \right)} \right) + z_{l - 1} \hfill \\ z_{l} &= MLP\left( {LN\left( {\hat{z}_{l} } \right)} \right) + \hat{z}_{l} \hfill \\ \end{aligned}$$

where $x_{p}^{i}$ represents the patches of input and $z_{0}$ represents the sequence of the image.

The key part of the transformer is MSA, which produces an attention weight map for the relationship between each pixel in every channel head. However, the standard MSA has two problems: first, the cost of computation to generate the attention weight map is quadratically related to the number of tokens, which represent the features of a certain area of the picture. For high-resolution medical images, several tokens will be generated, which will greatly increase the cost of computation. Second, when generating the attention weight map, the standard MSA does not involve the distance between two tokens. To address the aforementioned issues, axial attention and a weight mask are introduced to improve the network.

#### Axial attention

In a standard transformer, MSA generates the feature maps of Query (Q), Key (K), and Value (V) with the same dimensions by input and then decomposes them in the channel dimension according to the number of multi-heads. Finally, the attention map of each head is generated by Q × K and then multiplied by V. The operation includes the calculation of the correlation coefficient between each token, which will generate the quadratic computational complexity. Inspired by [21], in the P-transformer of this paper, Q and K are calculated separately according to H and W dimensions, and each dimension calculates the correlation coefficient $\hat{f}_{H} ,\hat{f}_{W}$ separately. The computational complexity is reduced, which can adapt to more tokens. Besides, the correlation matrix $\hat{f}$ only involves one dimension and reveals the attention inside the dimension. For example, $\hat{f}_{{H\left( {i,j} \right)}}$ represents the relation between the indexes $i,j$ in the H dimension.

**Fig. 2:**
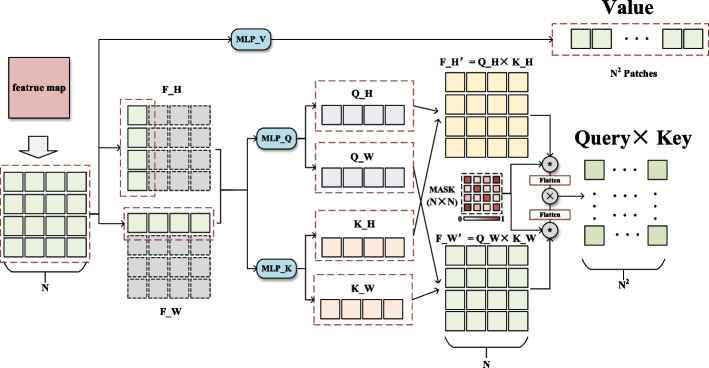
The detailed illustration of axial mask attention in P-transformer on the input feature map

As shown in Fig. 2, the detailed operations are as follows: after the input is divided into different tokens, take the mean value in the two dimensions of H and W, respectively, and then conduct the subsequent multi-head attention calculation. Before multiplying with V, the H and W dimensions are reconnected by matrix operation. The process can be represented by the following:

2 $$\begin{aligned} f_{H}& = Avg_{H} \left( {resize\left( {z_{l} } \right)} \right) \hfill \\ f_{W}& = Avg_{W} \left( {resize\left( {z_{l} } \right)} \right) \hfill \\ \hat{f}_{H} &= MLP_{Q} \left( {f_{H} } \right) \times MLP_{K} \left( {f_{H} } \right) \hfill \\ \hat{f}_{W} &= MLP_{Q} \left( {f_{W} } \right) \times MLP_{K} \left( {f_{W} } \right) \hfill \\ Q \times K &= flatten\left( {\hat{f}_{H} } \right) \times flatten\left( {\hat{f}_{W} } \right) \hfill \\ \end{aligned}$$

where $Avg$ represents the avg-pooling layer, $f,\hat{f}$ represents the axial feature in the process, and $\times$ represents matrix multiplication.

#### Attention weight mask

In a standard transformer, after obtaining the self-attention weight, MSA multiplies it with the V matrix for attention weighting. The self-attention feature map represents the relationship between every two tokens. The value of the activation is not concerned with the distance between tokens, indicating that the standard MSA will process all tokens equally; however, the correlation between tokens that are close or even adjacent can be better represented by the convolution layer. In this paper, we designed a parallel feature extraction method on the feature map to model the long-distance and local feature relationships separately using Vit and CNN-based models simultaneously. We hope that MSA can pay more attention to the relationship between long-distance tokens and reduce the correlation activation of short-distance positions. Therefore, we added a mask in the operation of axial attention ($\hat{f}_{H} ,\hat{f}_{W}$) to suppress the correlation activation of short-distance positions through a function. Take $\hat{f}_{{H\left( {i,j} \right)}}$ as an example; it represents the weight inside the H dimension and contains distance information in the $i,j$ index, which can be represented as $\left| {i - j} \right|$. Attention to short distances should be weakened to reduce similarity with CNNs.

In the axial attention calculation, the axial channel attention mask is generated. We used a weight distribution mask generated by a function for weight reassignment. The function sets weights in different positions according to their distances. Positions with close distances have smaller weights, and vice versa, up to the maximum value. The weight distribution map takes the diagonal line as the center line and increases from small to large on both sides, indicating that the module assigns more attention weights to distant features. The calculation of the weight distribution mask is as follows:

3 $$\begin{aligned} &Mask_{i,j} = \left\{ {\begin{array}{*{20}c} 1 & {i = j} \\ {k\left( {\left| {i - j} \right|} \right)^{2} - ka^{2} + 1} & {0 < \left| {i - j} \right| < a} \\ 1 & {\left| {i - j} \right| \ge a} \\ \end{array} } \right. \hfill \\ & Q \times K = flatten\left( {Mask \cdot \hat{f}_{H} } \right) \times flatten\left( {Mask \cdot \hat{f}_{W} } \right) \hfill \\ \end{aligned}$$

where $Mask_{i,j}$ represents the element of the weight distribution mask, $i,j$ represents the position, $\cdot$ represents dot multiplication, $k$ is a hyper parameter that was set to 0.5 in advance, and $a$ was set to half of the mask size.

#### ResBlock

In the parallel transformer structure proposed in this paper, another branch is the feature extraction network based on convolution. In our work, we chose Resnet-50 [22] as the backbone, which has five stages, and each stage is composed of multiple residual modules. Similar to TransUNet, which benefits from the excellent low-level feature extraction ability of the convolutional network, our network first performs a feature aggregation operation preliminarily on the input features through two stages for subsequent processing. In our proposed P-Transformer, each branch of the convolutional model consists of a stage in Resnet-50 to extract local correlation features. Benefiting from the local receptive field of the convolutional network, this branch mainly extracts the context information between close-range tokens and complements another transformer-based branch. The two branches extract the feature maps at different scales on the same input token sequence and generate outputs as the inputs of the subsequent feature fusion module.

### Global local fusing

After obtaining the two encoded features of different branches, we proposed a GLF module for efficient aggregation between features. We designed the GLF hoping that the global features extracted by the transform branch can strengthen or weaken the local features based on the CNN branch. Inspired by the Convolutional Block Attention Module(CBAM) [23], we first generated the spatial attention (SA) matrix by spatial average pooling and convolution layer of global features, and dot multiplying with the local features. We can filter and enhance the local features in the spatial dimension, and remove redundant information and noise simultaneously. Then, we generated the channel attention (CA) through channel average pooling and full connection layer in parallel, and filtered the local features on the channel dimension. In conclusion, the global features are used to guide and enhance the local features to realize the interaction between features. Besides, the global features and the adjusted local features are compressed in the channel dimension through a CNN separately and concate the outputs to generate the final features. Finally, a CNN is used to produce the outputs.

4 $$\begin{aligned} A_{s} &= SpatialAttention\left( {f_{global} } \right) \hfill \\ A_{c} &= ChannelAttention\left( {f_{global} } \right) \hfill \\ f_{out} &= conv\left( {cat\left( {conv\left( {f_{global} } \right),f_{local} \cdot A_{s} \cdot A_{c} } \right)} \right) \hfill \\ \end{aligned}$$

where $f_{local} ,f_{global}$ represents local and global features, respectively, $cat$ indicates a stack operation, and $A_{c} ,A_{s}$ denote channel and spatial attention, respectively.

### Edge information in loss function

During the training phase, P-TransUNet uses an end-to-end training method. We have used binary cross-entropy loss $L_{BCE}$ and dice loss $L_{Dice}$. The calculation formulas are as follows:

5 $$\begin{aligned} L_{BCE}& = - \sum\limits_{i = 1}^{n} {(y_{i} log(p_{i} ) + (1 - y_{i} )log(1 - p_{i} ))} \hfill \\ L_{Dice} &= 1 - \frac{{\sum\nolimits_{i = 1}^{n} {y_{i} p_{i} + \varepsilon } }}{{\sum\nolimits_{i = 1}^{n} {(y_{i} + p_{i} ) + \varepsilon } }} \hfill \\ \end{aligned}$$

where n is the total number of pixels in each image, $y_{i}$ represents the ground-truth value of the $i_{th}$ pixel, and $p_{i}$ represents the confidence score of the $i_{th}$ pixel in the prediction results.

Many studies have indicated the importance of edge information for generating a clear prediction in segmentation tasks. Similarly, for medical images with fuzzy edges and similar shapes, we also introduced edge information, as shown in Fig. 1. For a sample mask, we first extracted its edge mask using the Canny operator with a large threshold range to comprehensively extract edge information. Generally, the edge of the image only occupies a small part of the mask pixels, which leads to the imbalance of positive and negative samples. Therefore, we introduced Ohem loss[24] for edge masks, which only calculates the loss of part pixels. In detail, we first produced the loss of each pixel by prediction and ground truth, and chose some of the pixels with high loss to calculate the final loss. Ohem loss is more likely to collect these misclassified small samples for loss calculation, which is conducive to alleviating the problem of sample imbalance.

Except for the commonly used Dice and CE losses, the loss function used in training includes the introduced edge loss. The ground truth of the edge is produced by the mask image through the Canny operator in advance. Because of the imbalance of pixels in the edge mask, we introduced Ohem loss to reduce the impact of the imbalance of positive and negative samples. Therefore, the loss function finally used in this paper is as follows:

6 $$L_{total} = \alpha L_{CE} \left( {G,P} \right) + \beta L_{Dice} \left( {G,P} \right) + \gamma L_{Ohem} \left( {G_{edge} ,P_{edge} } \right)$$

where $\alpha$, $\beta$ and $\gamma$ are set to 0.5, 0.3, and 0.2, respectively; $G,G_{edge}$ represent the ground truth of each image and its edge, respectively, and $P,P_{edge}$ represents prediction of each image and its edge, respectively.

## Experimental analysis

In this section, we conducted some experiments to compare our proposed model with SOTA methods in four segmentations datasets.

### Description of datasets

#### Polyp segmentation

For the polyp segmentation task, we selected two public polyp datasets named Kvasir [25] and CVC-ClinicDB [26], which can be publicly accessed and downloaded. The Kvasir-SEG dataset collected 1,000 preprocessed polyp images, and the lesion mask was drawn by several medical experts. Each image in the Kvasir-SEG dataset can contain multiple polyps. Similar to [27], we randomly split the dataset into the training, test, and validation sets at an 8:1:1 ratio. The CVC-ClinicDB dataset comprises 612 randomly selected video frames from colonoscopic videos provided by the Barcelona Hospital in Spain. Each image contains only one polyp and has been marked by experts. We still split them by the same ratio as Kvasir.

#### GLAnd Segmentation (GLAS) dataset

GLAS datasets come from a competition in 2015, which provides images of hematoxylin and eosin (H&E)-stained slides to perform gland segmentation in histology images. GLAS contains 165 images with different resolutions. According to a study on GLAS [28], we classified 85 images as the training set and 80 images as the test set.

#### 2018 data science bowl (DSB)

2018 DSB is from a segmentation challenge and is used to find the nuclei in divergence [29]. 2018 DSB contains 670 images, and we split the dataset into three groups—80% for training, 10% for validation, and 10% for testing—according to the settings in [30].

### Evaluation metrics

To evaluate the proposed P-Transformer model, we used four standard evaluation metrics to compare with other SOTA methods. The evaluation metrics we used included Dice Coefficient (Dice or F1), Intersection over Union (IoU), Precision, and Recall, which are related to the confusion matrix values of the experimental results. There are four types of values in the confusion matrix, namely, true-positive (TP), true-negative (TN), false-positive (FP), and false-negative (FN) values. The calculated method of standard evaluation metrics is as follows:

7 $$\begin{aligned} Dice &= \frac{2 \times TP}{{2 \times TP + FP + FN}} \hfill \\ IoU &= \frac{TP}{{TP + FP + FN}} \hfill \\ Precision &= \frac{TP}{{TP + FP}} \hfill \\ Recall &= \frac{TP}{{TP + FN}} \hfill \\ \end{aligned}$$

### Implementation details

All models are built using the PyTorch framework and trained on an NVIDIA 3090 with the memory of 24 GB. We used the SGD optimizer with an initial learning rate of 0.01, a momentum of 0.9, and a weight decay of 0.0001, with Cosine Annealing warm restart schedule for more effective training. During the training, we set the batch size to 4 and the max epochs to 150. Each training saved the best model for testing.

In the training process, we resized all images to 512 × 512 for the experiment and various data enhancement technologies were introduced to expand the dataset. First, the images were processed using common sample enhancement techniques: random rotation, random horizontal and vertical inversion, clipping, and random elastic deformation. Additionally, one of the following methods is randomly selected to generate the final inputs: cut out, course dropout, grid destruction, and grid dropout.

### Results

#### Comparison on Kvasir-SEG

**Fig. 3:**
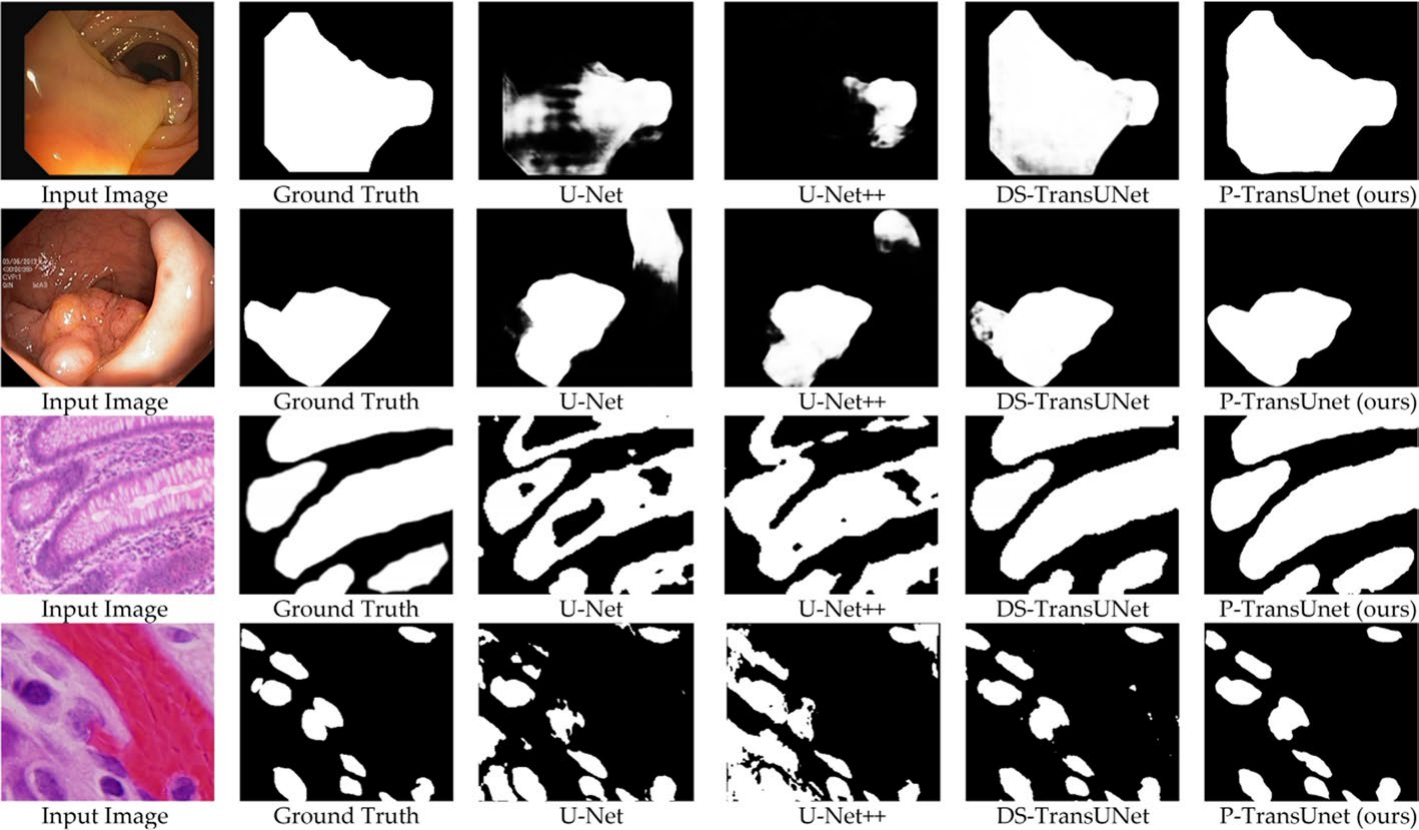
Qualitative results of P-TransUNet on four medical image segmentation datasets compared with other methods from [20]. From top to bottom: Kvasir, CVC-ClinicDB, GLAS, DSB

In Table 1, which only involves the Kvasir dataset, we can see that our P-TransUNet outperforms other methods on mDice by 93.52% and Recall by 93.89%. On mIoU and Precision, our method ranked second with 88.93% and 93.79% behind FCBFormer. The results demonstrated that our method improved mDice and Recall by 1.17% and 0.88%, which are valued to decrease the misdiagnosis rate in clinical practice. As shown in the visualization results (Fig. 3), our method can distinguish polyps with fuzzy edges, which have similar color and structure to normal tissue, which caused misdiagnosis using other methods.

**Table 1:** Comparisons with the state-of-the-art baselines on the Kvasir-SEG dataset

Method	mDice	mIou	Recall	Precision
DoubleU-Net [19]	0.8130	0.7330	0.8400	0.8610
ResUNet++ [31]	0.8133	0.7927	0.8774	0.7064
U-Net [5]	0.8180	0.7460	0.6306	0.9222
FCN [32]	0.8310	0.7370	0.8350	0.8820
DDANet [33]	0.8576	0.7800	0.8880	0.8643
FANet [34]	0.8803	0.8100	0.9060	0.9010
U-Net++ [6]	0.9032	0.8473	0.8923	0.8945
TransUNet [15]	0.9130	0.8570	–	–
DS-TransUNet [20]	0.9130	0.8592	0.9360	0.9164
MSRF-Net [35]	0.9217	**0.8914**	0.9198	**0.9666**
FCBFormer [36]	0.9235	0.8757	0.9301	0.9306
Our method	**0.9352**	0.8893	**0.9389**	0.9379

Terms: The “−” denotes that the corresponding result is not provided. For each column, the best results are highlighted

#### Comparison on CVC-ClinicDB

In Table 2, which only uses the CVC-ClinicDB dataset, the results showed that our P-TransUNet achieves SOTA on all metrics compared with other methods. Specifically, our method improved mDice, mIoU, Recall, and Precision by 1.32%, 1.22%, 0.62%, and 1.25% compared with FCBFormer. The visualization in Fig. 3 demonstrates that P-TransUNet can segment a large area of polyps more accurately than the previous method.

**Table 2:** Comparisons with the state-of-the-art baselines on the CVC-clinicDB dataset

Method	mDice	mIou	Recall	Precision
U-Net [5]	0.8781	0.7881	0.7865	0.9329
Deeplabv3+ [37]	0.8897	0.8706	0.9251	0.9366
PraNet [30]	0.8990	0.8490	–	–
U-Net++ [6]	0.9035	0.8637	0.9175	0.8564
ResUNet++ [31]	0.9199	0.8892	0.9391	0.8445
TransUNet [15]	0.9350	0.8870	–	–
FANet [34]	0.9355	0.8937	0.9339	0.9401
DS-TransUNet [20]	0.9422	0.8939	0.9500	0.9369
FCBFormer [36]	0.9461	0.9020	0.9502	0.9412
Our method	**0.9593**	**0.9142**	**0.9564**	**0.9537**

Terms. The “−” denotes that the corresponding result is not provided. For each column, the best results are highlighted

#### Comparison on 2018 data science bowl

The quantitative result of our network on 2018 DSB is shown in Table 3. The results showed that our proposed network P-TransUNet outperformed other SOTA methods on all metrics. Compared with FCBFormer, P-TransUNet improved mDice by 1.18%, mIoU by 1.48%, Recall by 0.84%, and Precision by 1.54%. From the qualitative results in Table 3, we can conclude that our P-TransUNet could find the position of cell nuclei more accurately and generate a clearer segmentation prediction on small samples.

**Table 3:** Comparisons with the state-of-the-art baselines on the 2018 data science bowl (DSB) dataset

Method	mDice	mIou	Recall	Precision
U-Net [5]	0.7573	0.9077	–	–
PraNet [30]	0.8103	0.7108	0.8062	0.8231
Deeplabv3 [37]	0.8857	0.8367	0.9141	0.9081
U-Net + + [6]	0.8853	0.8906	0.8862	0.8628
ResUNet [38]	0.8991	0.8244	0.9000	0.9084
Attention U-Net [11]	0.9083	**0.9103**	–	0.9161
TransUNet [15]	0.9178	0.8648	0.9023	0.8936
TransAttUnet [39]	0.9162	0.8498	0.9185	0.9193
DS-TransUNet [20]	0.9219	0.8612	0.9378	0.9124
MSRF-Net [35]	0.9224	0.8534	0.9402	0.9022
FCBFormer [36]	0.9245	0.8727	0.9379	0.9083
Our method	**0.9363**	0.8875	**0.9463**	**0.9237**

Terms: The “–” denotes that the corresponding result is not provided. For each column, the best results are highlighted

#### Comparison on GLAS

Based on the results on GLAS in Table 4, we can observe that the proposed P-TransUNet achieved better performance than previous SOTA methods on all metrics. Our model produced 89.22% on mDice, 81.24% on mIoU, 89.33% on Recall, and 89.57% on Precision with an improvement of 0.85%, 1.16%, 0.14%, and 1.08% compared with the leading SOTA method TransAttUnet. As shown in Fig. 3, the visualization result proved that our method can generate smoother segmentation results, particularly for samples with a vague edge.

**Table 4:** Comparisons with the state-of-the-art baselines on the GLAS dataset

Method	mDice	mIou	Recall	Precision
U-Net [5]	0.7976	0.6763	–	–
ResUNet [38]	0.8088	0.6911	0.8511	0.8001
MedT [9]	0.8102	0.6961	–	–
U-Net++ [6]	0.8245	0.7023	0.8324	0.8179
Attention U-Net [11]	0.8159	0.7006	–	–
TransUNet [15]	0.8634	0.7736	0.8573	0.8268
DS-TransUNet [20]	0.8719	0.7845	–	–
TransAttUnet [39]	0.8837	0.8008	0.8919	0.8849
FCBFormer [36]	0.8745	0.7903	0.8786	0.8523
Our method	**0.8922**	**0.8124**	**0.8933**	**0.8957**

Terms: The “–” denotes that the corresponding result is not provided. For each column, the best results are highlighted

## Generalization and discussion

In medical imaging, generalization ability refers to the robustness of the algorithm on different datasets. This paper used Kvasir-SEG for model training and then CVC-ClinicDB for tests. Similarly, we also exchanged datasets for research, that is, model training on CVC-ClinicDB and testing on Kvasir-SEG. Tables 5 and 6 show the results of the generalization experiments. Furthermore, we conducted ablation studies to explore the effectiveness of the proposed modules.

**Table 5:** Generalizability results of the models trained on Kvasir-SEG and tested on CVC-clinicDB

Method	mDice	mIoU	Recall	Precision
U-Net [5]	0.6302	0.5015	0.5612	0.8249
U-Net++ [6]	0.4267	0.3623	0.4337	0.6877
Deeplabv3 + Xception [37]	0.6509	0.5385	0.6251	0.7947
Deeplabv3 + Mobile [37]	0.6303	0.4825	0.5957	0.7173
HRNetSmallv2 [40]	0.6428	0.5513	0.6811	0.7253
HRNet [40]	0.7901	0.6953	0.8796	0.7694
MSRF-Net [35]	0.7921	0.6498	**0.9001**	0.7000
Our method	**0.8462**	**0.7584**	0.8364	**0.8681**

**Table 6:** Generalizability results of the models trained on CVC-clinicDB and tested on Kvasir-SEG

Method	mDice	mIou	Recall	Pre	Flop
Base(resblock)	0.8713	0.8056	0.8964	0.8665	18.8G
Base + T	0.9061	0.8376	0.9272	0.8993	47.6G
Base + P-transformer	0.9253	0.8652	0.9354	0.9190	35.4G
Base + T + GLF	0.9161	0.8479	0.9304	0.9129	50.8G
Base + T + edge	0.9123	0.8474	0.9296	0.9045	49.8G
Base + P-transformer + GLF + edge	**0.9352**	**0.8893**	**0.9389**	**0.9379**	**42.6G**

### Generalizability results

To study the generalization performance of our model in different datasets of the same type, we performed generalization experiments on two gastric polyp datasets, as shown in Tables 5 and 6. According to the split of experiments, we trained with the Kvasir and CVC training sets, respectively, and then tested on the CVC and Kvasir test sets instead. Compared with previous studies, our P-TransUNet model achieved better performance in two generalization experiments on mDice and mIoU, but has a deficiency in Recall and Precision. The results showed that our model could extract more descriptive features at a high level and had a stronger ability to generalize image data of the same types.

### Ablation study

To explore the impact of each proposed module in this paper, we conducted an ablation study on the Kvasir dataset. Specifically, we used ResBlock to build the encoder as the baseline and then added a transformer to verify the effectiveness of the parallel structure. In the experimental part, we added the proposed P-Transformer, GLF, and edge loss to perform experiments with quantitative analysis. As shown in Table 7, the results indicated that the model with three improvements performed best on four indicators.

**Table 7:** Ablation study of P-TransUNet on the Kvasir-SEG dataset for each column

Method	mDice	mIoU	Recall	Precision
U-Net [5]	0.5621	0.405	0.4364	0.8466
U-Net++ [6]	0.6783	0.5494	0.7311	0.6885
HRNet-Smallv2 [40]	0.2107	0.1363	0.2038	0.3347
HRNet [40]	0.2349	0.2461	0.3372	0.1523
Deeplabv3 + Xception [37]	0.6746	0.5327	0.6296	0.7757
Deeplabv3 + Mobile [37]	0.6474	0.5098	0.6632	0.6878
MSRF-Net [35]	0.7575	0.6337	0.7197	**0.8414**
Our method	**0.7911**	**0.6876**	**0.8409**	0.7825

The best results are highlighted. (T means transformer)

*Effects of P-transformer*: To solve the training problem of the standard transformer, we proposed an improved version that includes axial attention and an attention weight mask. Experiments were conducted to evaluate the effectiveness of the module. The experimental results showed that the adjusted P-transformer can improve the performance of all evaluation indexes, particularly on mDice and mIoU, by 1.92% and 2.76%, respectively. It was proven that the P-transformer can effectively guide parallel branches to extract global and local information, and reduce information redundancy. Simultaneously, compared with the direct introduction of the P-transformer, the module can reduce the computational complexity from 47.6 G to 35.4 G, approximately 25% reduction, and obtain greater performance improvement due to the division of labor in feature modeling. Our proposed model also benefits from this structure, which further improves the identification ability of the network under the premise of reducing parameters.

*Effects of GLF*: For the fusion of features extracted from the double-branch structure, we proposed a GLF module to fuse global and local features. We added this module to the baseline and achieved a performance improvement of 1% on mDice, 1.03% on mIoU, and 1.36% on Precision. From the quantitative results, we can conclude that the GLF module can effectively fuse the long-distance and local feature information, and improve the feature modeling ability of the model. A unique capability of GLF is feature fusing, and additional supervision information is required to guide the feature extraction.

*Effects of edge loss*: To solve the problem of lacking edge information in the medical imaging field, we introduced edge loss into the model to guide the network to focus on the edge of the regions of interest. We added edge loss to the network and conducted experiments. The baseline added with edge loss achieved performance improvement by 0.62% and 0.98% on mDice and mIoU, respectively. The quantitative analysis of the experimental results showed that the guidance edge information is conducive to the network to extract the distinguishing features to improve segmentation performance.

### Model visualization

**Fig. 4:**
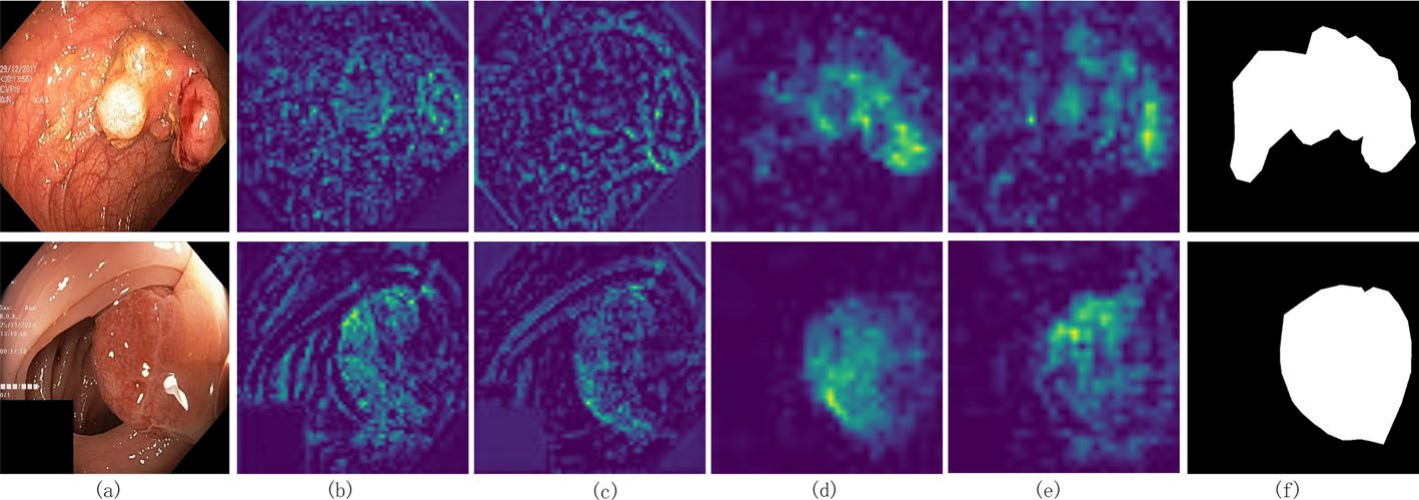
Feature visualization diagram on Kvasir dataset. Where **a** is the input image, **b** and **d** are the output of the encoder GLF layer of the proposed model, **c** and **e** are the output of the corresponding layer of the basic network, and **f** is the groundtruth

This section visualizes the output of the encoder stage of the model and the basic model (ResBlock + T) to show its aggregation ability on global information. As shown in Fig. 4, columns 2 and 4 are the output features of the first and second encoder layers, respectively, and columns 3 and 5 are the outputs of the encoder layer corresponding to the basic network. By comparing the visualization results, it was found that our model can effectively aggregate global and local information, and strengthen the attention of the network to the lesion area. Furthermore, because of the aggregation of multiscale comprehensive information, the high magic heart can also smoothen the activation of the lesion area, reduce the misjudgment of the internal small range, and better conform to the common shape characteristics of the lesion. In comparison, the P-TransUNet proposed in this paper can better mine the significant features of the lesion area at different scales.

## Conclusion

In this work, we proposed the P-TransUNet that is based on the U-shaped encoder–decoder framework for medical image segmentation tasks. Our P-TransUNet uses P-Transformer blocks to obtain the global and local features of images in parallel. Furthermore, we improved the standard structure of the transformer by axial attention and an attention weight mask to extract long-range features. Then, an attention-based GLF module is used for feature fusion. The GLF module adjusts the attention weight on the channel and spatial dimensions, and uses the residual module to fuse the features. Furthermore, we introduced edge loss in the training process to guide the network focus on the edge of the area of interest so that the model can learn the discriminating information between the target and the background area. The experiments on four datasets of multiple medical image segmentation tasks showed that our P-TransUNet outperforms other SOTA methods, and ablation experiments also proved the effectiveness of each module. In the future, we will focus on designing a more lightweight structure based on the transformer for embedded devices in the clinic and on building a larger video dataset in the medical field for further research.

## Data Availability

All datasets used in this paper are publicly available. The Kvasir-SEG is publicly available at: https://datasets.simula.no//kvasir-seg/#download. The CVC-ClinicDB is publicly available at : https://polyp.grand-challenge.org/CVCClinicDB/. The 2018 Data Science Bowl is publicly available at : https://www.kaggle.com/competitions/data-science-bowl-2018/data. The GLAS is publicly available at: https://warwick.ac.uk/fac/cross_fac/tia/data/glascontest/download/.
